# SARS-CoV-2 (COVID-19) Vaccine Development and Production: An Ethical Way Forward

**DOI:** 10.1017/S096318012000047X

**Published:** 2020-06-05

**Authors:** KENNETH V. ISERSON

**Keywords:** COVID-19, SARS-CoV-2 virus, vaccines, vaccine testing, immunization

## Abstract

The world awaits a SARS-CoV-2 virus (i.e., COVID-19 disease) vaccine to keep the populace healthy, fully reopen their economies, and return their social and healthcare systems to “normal.” Vaccine safety and efficacy requires meticulous testing and oversight; this paper describes how despite grandiose public statements, the current vaccine development, testing, and production methods may prove to be ethically dubious, medically dangerous, and socially volatile. The basic moral concern is the potential danger to the health of human test subjects and, eventually, many vaccine recipients. This is further complicated by economic and political pressures to reduce government oversight on rushed vaccine testing and production, nationalistic distribution goals, and failure to plan for the widespread immunization needed to produce global herd immunity. As this paper asserts, the public must be better informed to assess promises about the novel vaccines being produced and to tolerate delays and uncertainty.

## Introduction

The world expects a SARS-CoV-2 vaccine (against the COVID-19 disease) to appear so that life can return to a near-normal condition. All social, economic, and healthcare system plans have built in such a discovery. Vaccine safety and efficacy requires meticulous testing and oversight; under the current development, testing, and production schedules, however, vaccines may prove to be ethically dubious, medically dangerous, and socially volatile. The purpose of this paper is to better inform the public to be able to assess vaccine promises about the novel vaccines being produced and to tolerate delays and uncertainty.

Most experts agree that having a safe, effective, affordable, and widely available vaccine will be the only way to end the pandemic, both medically and socially. The pandemic’s medical end will come when about 70% of the world’s population—roughly 5.6 billion people—is immune, through either natural immunity or vaccination. To end the pandemic’s social effects, people will need confidence that they can again participate in their work and recreational activities without fear of contracting the disease. However, repeated promises of a rapidly produced vaccine, ethically and scientifically dubious routes being taken to develop a vaccine, and planned distribution systems favoring rich countries may strengthen the antivaccination movement, ultimately lengthening, rather than shortening, the pandemic. We can overcome these deficiencies by making the entire process transparent to the public and the healthcare community. This entails providing consistent honest assessments of vaccine development progress, disseminating sophisticated pro-vaccination education, and developing an equitable distribution program.

## Vaccine Development

Producing vaccine for a new disease or for a disease for which a vaccine does not exist (i.e., novel vaccine) requires completing the same steps to ensure safety and efficacy that are required for other vaccines and most medications. The normal steps in vaccine development are: exploratory stage, preclinical (laboratory and animal testing) stage, clinical development (three separate human testing steps), regulatory review and approval, manufacturing, and quality control.^1^ All steps must succeed to produce a successful vaccine. It is analogous to running the bases in baseball. Even if you round all the bases, you must ultimately cross home plate safely (i.e., U.S. Food and Drug Administration [FDA] approval).

This is a complex and enormously expensive undertaking. In the United States, the National Institutes of Health’s (NIH) Accelerating COVID-19 Therapeutic Interventions and Vaccines (ACTV) initiative, the Warp Speed project, and the Coalition for Epidemic Preparedness Innovations are each leading separate efforts in conjunction with pharmaceutical manufacturers to rapidly produce a vaccine. The U.S. programs have announced their intention to provide the U.S. population with their products before anyone else. The World Health Organization (WHO) and other groups are working through the Access to COVID-19 Tools Accelerator program to coordinate vaccine production and equitable global access. Other pharmaceutical companies, especially in India and China, are moving forward alone.^2^

### Vaccine Testing

Candidate vaccines developed in the laboratory normally must demonstrate that they can safely provide long-term immunity, first in laboratory animals, and then in progressively larger groups of human volunteers. Many current SARS-CoV-2 vaccine developers are skipping, abbreviating, or dangerously modifying these steps. The U.S. Warp Speed project has said that it is doing animal testing of its eight candidate vaccines in parallel with human testing.^3^ Other groups are using methods that have never produced a successful vaccine, such as messenger RNA encoding the coronavirus surface protein or using an adenovirus to deliver the same protein’s gene.^4^ Such ethically and medically dubious shortcuts will eventually engender fear and mistrust in potential vaccine recipients, especially because few people are aware of how these procedural changes may affect the vaccine’s safety and efficacy. When they ultimately find out, this may dissuade many people from being immunized.

### Animal Testing

An initial and vital step in designing vaccine studies is to define the safety, efficacy, and other criteria, called a “target product profile” (TPP), that must be met for the test vaccine to progress to the next stage. Most new medications fail to meet their targets during testing (Table 1). A major TPP is assuring the compatibility and stability of the vaccine’s adjuvant (used to improve the immune response) and antigen. This is normally done through in vivo tests in animals, and can take months, if not years, to complete. If the results demonstrate that the vaccine is dangerous, it does not move on to human testing. For example, animal testing of some non-COVID-a9 coronavirus vaccines has shown an increased risk of the animals getting the disease rather than preventing it.^5^ ^,^^6^ Other animal tests reveal that vaccines are ineffective; that is, they do not trigger antibody production. In fact, medications often fail to demonstrate that they can successfully modify the disease or health concern they are designed to address. Only about 12% of pharmaceutical candidates that go through this rigorous evaluation, including vaccines, make the transition from the laboratory to clinical trials.^7^ ^,^^8^

**Table 1.** Percent of New Drugs Failing Human Testing^9^

37% of drugs entering Phase I trials fail.
69% of drugs entering Phase II trials fail.
No data available for human-challenge studies trials, since they are so rare.
42% of drugs entering Phase III trials fail.
15% of drugs that finish Phase III trials and are submitted for an FDA New Drug Application fail to get approved.

### Human Vaccine Trials

If a candidate vaccine meets its TPPs in animal tests, human testing begins. Such clinical trials follow established guidelines from the European Medicines Agency, the WHO, the FDA, and other national and supranational bodies. Clinical testing progressively assesses the vaccine’s safety and efficacy while producing the least foreseeable harm in test subjects.

The first tests (Phase I) are done with a small group (20–100) of healthy volunteers. This phase usually lasts several months, during which scientists determine the vaccine’s safety and the effect of different vaccine doses on side effects and efficacy (antibody and T-cell production).^10^ ^,^^11^ In the current rush to produce a vaccine, some Phase I trials have lasted no more than 3 weeks before being rushed into much larger Phase II trials (normally using hundreds to thousands of volunteer human subjects).^12^ This interval is far too brief to assess whether TPPs have been achieved. It is reasonable to assume that many of these Phase II vaccines will be unsafe or ineffective since, in recent years, only about 10% of all drugs entering Phase I trials eventually gained FDA approval.^13^

#### Ethics of abbreviating animal and human testing and the government approval methods.

The basic moral concern is the potential danger to the health of human test subjects and, eventually, the large number of vaccine recipients. In truth, the risk–benefit ratio is acceptable for fully informed volunteer test subjects, even when they are knowingly receiving a potentially lethal virus. Without transparency to the public, however, it is ethically dubious to expose the public to the possible risk of harm from unsuspected side effects or ineffectiveness; this may outweigh any potential benefits of abbreviated vaccine production. Any such results will feed the inherent distrust of vaccination among the antivaccination community, diminishing the chance to ultimately immunize at least 70% of the world to achieve herd immunity. To ameliorate this issue, we ought, at the least, to publicly describe the risks human-challenge study (HCS) subjects are taking, make the criteria for vaccine approval transparent to the public and healthcare community, and admit what still is not known about any vaccine before it is released, including the chance of recipients having complications or not being immune to SARS-CoV-2.

## Human Trial Subjects

Little has been said publicly about the volunteer subjects being used in SARS-CoV-2 vaccine trials. While Institutional Review Boards normally monitor how trial subjects are selected, consented, and protected, it is unclear what ethical oversight if any is in place for many of the current trials. In some cases, the process has been so rapid that it is unlikely that much monitoring has been done.

How will the public react if, given the omission of so many safety steps in the process, some trial subjects become ill (ineffective vaccine) or die (unsafe vaccine)? If the vaccines merely fail to provide protection, the population may get “vaccine fatigue,” tiring of constant promises, and not wish to participate in trials. If deaths occur among vaccine trial subjects, we should expect that volunteer enthusiasm for other vaccine trials will diminish, especially after the publication of exposés that detail the process’s failings. The public also may be wary of accepting a vaccine, even if authorities say that it is safe and effective, given the mixed messages issued during this pandemic (e.g., advice to ingest Clorox and use hydroxychloroquine). Also, since only 69% of medications undergoing Phase II trials meet their TPPs (Table 1) and only about 10% of new drugs eventually gain FDA approval, the first successful SARS-CoV-2 vaccine will most likely be the 42nd or even the 90th one to complete human testing. (About 110 vaccines are in development as of mid-2020.)

The normal trial method for both Phase II and the subsequent, generally much larger Phase III tests is randomized control trials (RCTs). This takes significant time as well as volunteer subjects’ willingness to possibly receive the placebo. So much publicity now surrounds the test vaccines that obtaining valid informed consent may be problematic. Magical thinking (“my test vaccine will work”) will invariably attract participants who may enroll in the trial to be a hero: a member of the test of a vaccine that could save the world from SARS-CoV2 and prioritize their country for receiving the vaccine.^14^

While RCTs are considered to be the most reliable method to assure that the resulting vaccine is safe and effective, because these trials take so long, it is highly unlikely that most novel SARS-CoV-2 vaccine trials will use RCTs with standard TPPs (i.e., proving long-lasting antibody production, minimal side effects, and appropriate dosing schedule).

### 

#### Ethics of overstating the chance of obtaining a safe and effective vaccine in a short period of time.

Even if all testing and manufacturing steps work well, producing a safe and effective SARS-CoV-2 vaccine will probably be a long process. Because trust is essential to maintain viable leadership, truth telling is a key element in the fight against COVID-19, while dishonesty and hyperbole will undermine all other efforts. This includes full disclosure about uncertainty around vaccine availability, which will greatly disappoint for those unfamiliar with medical science. For politicians, it will be ego challenging. Pharmaceutical company stockholders will fear for the enormous investments being made. On balance, the public will tolerate the truth much better than repeated unfulfilled promises. Thus, we ought to clearly and consistently state that no one knows when a safe, effective SARS-CoV-2 vaccine will be available, although we are using all available resources to make that happen.

### Infecting Trial Subjects, HCS

Because of the short timeline many vaccine developers have announced, it is likely that they will be using an uncommon abbreviated testing method in which a small group of healthy volunteers are all randomized, given either an experimental vaccine or a placebo, and then infected with the pathogen. This method, called HCS, has a long history, the most famous use being in 1796 when Edward Jenner infected a young boy with smallpox after inoculating him with Cowpox, an experimental vaccine. HCS was used to develop the typhoid and cholera vaccines, but also has been associated with ethically suspect research that helped to stimulate development of the Nuremberg Code (1946) and the Declaration of Helsinki (1964).^15–17^

Over the past few decades, the use of HCS has markedly increased, especially in low- to middle-income countries. The benefit of such trials is that they use many fewer test subjects and are conducted over a much shorter time period. Normal Phase III trials often involve thousands of subjects; HCS trials may enroll fewer than 100 while still providing the information necessary to determine if the vaccine is safe and effective.^18^ Although physicians participating in HCS trials seemingly breach the basic professional principle to “do no harm,” they enter such research to stop or prevent worldwide pandemics.^19^ Nonetheless, some argue that HCS trials, with their inherent risk to participants, can only be performed in “treatable or self-limiting diseases where no irreversible pathology is known to occur”—certainly not the case with COVID-19.^20^ ^,^^21^

Because it is presumed that many developers will use this method for the SARS-CoV-2 vaccine trials (and ACTV has announced that they are considering it^22^), the WHO has developed a list of necessary criteria to address ethical issues associated with such studies (Table 2).^23^ These criteria involve concerns about the infection’s risks to participants, the research staff, and the community.

**Table 2.** WHO Requirements for HCS Trials^24^

• Strong scientific justification.
• The potential benefits outweigh the risks.
• Researchers must consult and closely coordinate with the public, experts, funders, regulators, and policy makers.
• The studies are conducted at sites that can maintain the highest scientific, clinical, and ethical standards.
• Participant selection criteria limit and minimize risk.
• Studies are reviewed by specialized independent committees.
• Studies must use rigorous informed consent.

#### Ethics of HCS Trials

It is unclear whether, in their haste to produce a viable vaccine, most researchers will follow the WHO criteria for ethical HCS trials, although even these standards may not be adequate to protect research subjects. Following these protocols may reduce negative public reaction, but if significant numbers of people were to die as a result of taking part in vaccine studies following HCS protocols, then a widespread public reaction should be anticipated. As Hope and McMillan wrote, “The idea of giving a healthy person a disease is so alien to the public’s expectations of medicine and medical research that it is vital to conduct such studies within a well-considered and transparent set of guidelines and regulatory processes.”^25^

Doing HCS may raise additional ethical and legal questions. These include whether vaccine trial participants can volunteer for a potentially lethal experiment; whether physicians can violate their duty to do no harm; whether social and political pressure and general misinformation allows for true informed consent; and if this type of study conforms to the norm of “minimal risk” or “minimal harm” (generally undefined) described by most research guidelines.^26^

Yet, if actual volunteers (not military members, as has been suggested) receive enough information to give meaningful informed consent, and the risks and benefits are publicized in advance (instead of after complications or deaths), using this testing method will pass ethical muster with most people. In philosophy, the method is similar to the classic trolley dilemma, where one person may be sacrificed to save a group. In the SARS-CoV-2 vaccine trials, the reward–risk ratio is significantly higher.^27^

Not only will HCS provide a faster and less-expensive answer to whether the vaccine works, but it will also require many fewer individuals to be subjected to the risks from an experimental and possibly ineffective vaccine. The small number of participating volunteers will provide numerous benefits to the world, including saving tens or even hundreds of thousands of lives and hastening a return to normal social functioning, with its economic and public health benefits.^28^

## Subsequent Human Testing, Approval, and Manufacture

After successful Phase II trials, much larger Phase III trials follow, often lasting several years. If scientific experts agree that a vaccine’s test results meet all necessary criteria, they can permit it to be marketed. This complex process often requires additional data from manufacturers and, sometimes, additional trials. In the case of SARS-CoV-2 vaccines, regulators may not require Phase III trials if HCS meet whatever expectations they establish. In any case, with vaccine producers claiming that their vaccine will be ready for the public in 2021 or earlier, neither Phase II nor Phase III trials will last as long as normal.

Normally, only after a drug is approved does the pharmaceutical company begin production, a multimillion-dollar endeavor. Vaccine production, depending on the process used and whether a new plant must be built, may last months to years. Many companies (e.g., Serum in India, the U.S. Warp Speed project, Johnson & Johnson, and others) are already planning or have started SARS-CoV-2 vaccine production, even before trial results are available. Johnson & Johnson announced that it plans to begin producing 300 million doses each year, even though it will be months before they do the first human trials. Pfizer plans to have up to 20 million doses available in Fall 2020. They have stated that they are certain that government approval will be forthcoming. Moderna, one of the first companies to start human trials, is relying on governments to place large orders before the products are formally approved.^29^ In the United States, the FDA can grant an “Emergency Use Authorization” (EUA) to speed moving the vaccine to market. The assertion that governments will grant an EUA or equivalent before a vaccine is tested suggests both ethical and scientific breaches of faith.

No international agreement governs international vaccine distribution, and public health experts fear a repeat of the 2009 H1N1 swine-flu vaccine debacle, when wealthy countries left poorer countries with little supply during the pandemic.^30^ Providing equitable vaccine distribution will be difficult. It is vital, however, that the multiple institutions, funders, governments, and pharmaceutical companies involved in seeking a SARS-CoV-2 vaccine should agree to provide fair and equitable distribution throughout the globe *before* knowing which group will achieve success.^31^

The race for a vaccine is not an altruistic endeavor. With governments providing massive funding for many of these efforts, we should ask: Who owns any ultimately successful vaccines? A parallel and possibly more important question is who will own the technology (i.e., the patents) to produce the vaccine and similar products? Finally, how much will the vaccines cost and who will pay for them? Serum, the private Indian manufacturer, says that it plans on charging 1,000 rupees (~$13) per dose, but believes governments will provide it to their citizens without charge.^32^ No other group has ventured a guess on what its vaccine may cost, although DNA- and RNA-based vaccines are expected to cost far more than those using traditional methods.^33^ History suggests that vaccine costs may differ by market (e.g., pneumococcal and human papillomavirus vaccines), thus penalizing some populations. On a different note, if the COVID-19 pandemic suddenly ceases, as did SARS, will pharmaceutical companies be willing to step up during subsequent pandemics?

### Potential Public Response: Side Effects and Distribution

A foreseeable consequence to testing shortcuts will be the recognition of unwanted side effects once a vaccine is distributed to larger groups. Such discoveries are a major reason for conducting extended large Phase III (or postmarketing Phase IV) trials for new medications. With no warning, the occurrence of potentially serious, and possibly lethal, side effects is likely to enrage the public, the healthcare community, and the political establishment.

Complications while testing vaccines or medical complications after a vaccine is on the market will make people even more reluctant to accept a viable vaccine when it is finally available. That is assuming that it will be available to everyone, including the most vulnerable populations. While Serum announced that India will distribute their vaccine to citizens without charge, no one else has discussed who will pay for vaccinations. Serum has also affirmed that their country will receive the first supply and that the Indian government will decide which countries receive later batches; the U.S. Warp Speed project has said the same.^34^ ^,^^35^ Sanofi, a French company, said that its entire vaccine supply will go to the United States, although the French government says it will not permit that to happen.

Distributing a vaccine, even those known to work well (e.g., polio, Ebola, and measles), to enough people to decrease the risk of disease can be challenging. While many groups throughout the world will clamor to receive any new vaccine, there are those who will actively oppose its distribution. Often labeled “antivaxxers,” these groups oppose all vaccines due to their fear and distrust of science, governments, medicine, and other nationalities or racial groups. If vaccine “passports” are required to fly or to attend school, as has been suggested, this will further feed into these groups’ conspiracy theories. Such groups are devoted to their cause, expertly manipulate social media, and have a rapidly growing media presence opposing a SARS-CoV-2 vaccine.^36^ ^,^^37^ In some areas of the world, they are violent. This will limit the ability to distribute the vaccine to many groups and in many areas of the world, thus thwarting the opportunity to eliminate the virus or to develop herd immunity.

#### Ethics of keeping initial vaccine batches for the producing nation’s citizens without plans to supply the entire world, especially the least-developed populations.

Nations survive because, in times of need, generosity and supporting others is the norm in nearly every culture. The privilege of wealth or the good fortune to produce a vaccine engenders the responsibility to equitably share, rather than hoard resources. Pragmatically, as members of the world’s population, we have a moral duty to eliminate lethal diseases when possible, including among the least-advantaged populations. Any vaccine allocation decision made behind John Rawls’ veil of ignorance would result in equitable distribution.^38^ Since diseases do not recognize national borders, it is in everyone’s interest to widely distribute the vaccine. To address this issue, the world’s governments, vaccine producers and funders, and healthcare agencies should immediately devise a plan to equitably share all vaccines that are proven to be safe and effective.

While no one can predict how long it will take to produce a viable vaccine, even using these abbreviated testing methods, many of the potential ethical, social, and political problems raised in this paper can be mitigated by transparency. Some vaccine developers, such as the coalition led by the U.S. NIH, have publicly detailed many aspects of their process, at least in a format available to and understandable by biological scientists and healthcare professionals. What we now need is for all vaccine developers to do likewise: all private and government leaders involved in the process should use the news and social media in as sophisticated, persistent, and entertaining a manner as the antivaxxers are doing, to clearly explain in lay terms what they are doing, how long it may take, and the chances of any one developer producing an ultimately successful vaccine. Only when potential recipients understand the process’s complexities as well as their personal risks will they be able to tolerate the uncertainties in our near future.

## References

[1] Centers for Disease Control and Prevention. Vaccine testing approval process; available at https://www.cdc.gov/vaccines/basics/test-approve.html (last accessed 29 Apr 2020).

[2] Cohen J. Unveiling “Warp Speed,” the White House’s America-first push for a coronavirus vaccine. *Science Magazine* 2020 May 12; available at https://www.sciencemag.org/news/2020/05/unveiling-warp-speed-white-house-s-america-first-push-coronavirus-vaccine (last accessed 13 May 2020).

[3] See note 2, Cohen 2020.

[4] See note 2, Cohen 2020.

[5] Takano T, Yamada S, Doki T, Hohdatsu T. Pathogenesis of oral type I feline infectious peritonitis virus (FIPV) infection: Antibody-dependent enhancement infection of cats with type I FIPV via the oral route. Journal of Veterinary Medical Science 2019;81(6):911–5. 31019150 10.1292/jvms.18-0702 PMC6612493

[6] Kam YW, Kien F, Roberts A, Cheung YC, Lamirande EW, Vogel L, et al. Antibodies against trimeric S glycoprotein protect hamsters against SARS-CoV challenge despite their capacity to mediate FcγRII-dependent entry into B cells in vitro. Vaccine 2007;25(4):729–40. 17049691 10.1016/j.vaccine.2006.08.011 PMC7115629

[7] Singh K, Mehta S. The clinical development process for a novel preventive vaccine: An overview. Journal of Postgraduate Medicine 2016:62(1):4–11. 26732191 10.4103/0022-3859.173187 PMC4944327

[8] American Pharmaceutical Review. Preclinical development: The safety hurdle prior to human trials. *American Pharmaceutical Review* 2016 April 30; available at https://www.americanpharmaceuticalreview.com/Featured-Articles/187349-Preclinical-Development-The-Safety-Hurdle-Prior-to-Human-Trials/ (last accessed 15 May 2020).

[9] Biotechnology Innovation Organization, Biomedtracker, Amplion. As cited in: Thompson SA. How long will a vaccine really take? Available at https://www.nytimes.com/interactive/2020/04/30/opinion/coronavirus-covid-vaccine.html?campaign_id=57&emc=edit_ne_20200430&instance_id=18115&nl=evening-briefing&regi_id=88426177&segment_id=26333&te=1&user_id=698624f6bce25e600ce6522455a17609 (last accessed 30 Apr 2020).

[10] See note 1, Centers for Disease Control and Prevention.

[11] See note 7, Singh, Mehta 2016.

[12] Liu A. China's CanSino Bio advances COVID-19 vaccine into phase 2 on preliminary safety data. *FiercePharma* 2020 Apr 10; available at https://www.fiercepharma.com/vaccines/china-s-cansino-bio-advances-covid-19-vaccine-into-phase-2-preliminary-safety-data (last accessed 17 Apr 2020).

[13] See note 9, Thompson.

[14] Alenichev A. Ethics and etiquette in an emergency vaccine trial: The orchestration of compliance. Global Bioethics 2020;31(1):13–28. 32158366 10.1080/11287462.2020.1726591 PMC7048227

[15] Roestenberg M, Hoogerwerf MA, Ferreira DM, Mordmüller B, Yazdanbakhsh M. Experimental infection of human volunteers. The Lancet Infectious Diseases 2018;18(10):e312–22. 29891332 10.1016/S1473-3099(18)30177-4

[16] Jin C, Gibani MM, Moore M, Juel HB, Jones E, Meiring J, et al. Efficacy and immunogenicity of a Vi-tetanus toxoid conjugate vaccine in the prevention of typhoid fever using a controlled human infection model of Salmonella Typhi: A randomised controlled, phase 2b trial. Lancet 2017;390(10111):2472–80. 28965718 10.1016/S0140-6736(17)32149-9 PMC5720597

[17] Tacket CO, Cohen MB, Wasserman SS, Losonsky G, Livio S, Kotloff K, et al. Randomized, double-blind, placebo-controlled, multicentered trial of the efficacy of a single dose of live oral cholera vaccine CVD 103-HgR in preventing cholera following challenge with *Vibrio cholerae* O1 El tor inaba three months after vaccination. Infection and Immunity 1999;67(12):6341–5. 10569747 10.1128/iai.67.12.6341-6345.1999 PMC97039

[18] Jamrozik E, Selgelid MJ. Human infection challenge studies in endemic settings and/or low-income and middle-income countries: Key points of ethical consensus and controversy. Journal of Medical Ethics 2020. Published online May 2020. doi: 10.1136/medethics-2019-106001 PMC7476299 32381683

[19] Hope T, McMillan J. Challenge studies of human volunteers: Ethical issues. Journal of Medical Ethics 2004;30(1):110–6. 14872087 10.1136/jme.2003.004440 PMC1757139

[20] Miller FG, Grady C. The ethical challenge of infection-inducing challenge experiments. Clinical Infectious Diseases 2001;33(7):1028–33. 11528576 10.1086/322664

[21] See note 15, Roestenberg et al. 2018.

[22] See note 2, Cohen 2020.

[23] World Health Organization. Key criteria for the ethical acceptability of COVID-19 human challenge studies. 2020 May 6; available at https://apps.who.int/iris/bitstream/handle/10665/331976/WHO-2019-nCoV-Ethics_criteria-2020.1-eng.pdf (last accessed 12 May 2020).

[24] See note 23, World Health Organization 2020.

[25] See note 19, Hope, McMillan 2004.

[26] See note 19, Hope, McMillan 2004.

[27] Dahl FA, Oftedal G. Trolley dilemmas fail to predict ethical judgment in a hypothetical vaccination context. Journal of Empirical Research on Human Research Ethics 2019;14(1):23–32. 30382789 10.1177/1556264618808175

[28] See note 18, Jamrozik, Selgelid 2020.

[29] Rowland C, Johnson CY, Wan W. Even finding a covid-19 vaccine won’t be enough to end the pandemic. *Washington Post* 2020 May 14; available at www.washingtonpost.com/business/2020/05/11/coronavirus-vaccine-global-supply/ (last accessed 14 May 2020).

[30] See note 29, Rowland et al. 2020.

[31] Bollyky TJ, Gostin LO, Hamburg MA. The equitable distribution of COVID-19 therapeutics and vaccines. JAMA 2020. Published online May 7, 2020. doi:10.1001/jama.2020.6641 32379268

[32] Siddiqui Z, Heinrich M. India's Serum Institute to make millions of potential coronavirus vaccine doses. *New York Times* 2020 Apr 28; available at www.nytimes.com/reuters/2020/04/28/world/europe/28reuters-health-coronavirus-india-vaccine.html?campaign_id=154&emc=edit_cb_20200501&instance_id=18119&nl=coronavirus-briefing&regi_id=68748894&segment_id=26340&te=1&user_id=5c1b087964feba4034dec8f6ec3c49c7 (last accessed 30 Apr 2020).

[33] Akpan N. Why a coronavirus vaccine could take way longer than a year. *National Geographic* 2020 Apr 10; available at https://www.nationalgeographic.com/science/2020/04/why-coronavirus-vaccine-could-take-way-longer-than-a-year/?campaign_id=154&emc=edit_cb_20200501&instance_id=18119&nl=coronavirus-briefing&regi_id=68748894&segment_id=26340&te=1&user_id=5c1b087964feba4034dec8f6ec3c49c7 (last accessed 30 Apr 2020).

[34] See note 2, Cohen 2020.

[35] See note 32, Siddiqui, Heinrich 2020.

[36] Roose K. Get ready for a vaccine information war. *New York Times* 2020 May 13; available at www.nytimes.com/2020/05/13/technology/coronavirus-vaccine-disinformation.html?action=click&module=Spotlight&pgtype=Homepage (last accessed 13 May 2020).

[37] Johnson NF, Velásquez N, Restrepo NJ, Leahy R, Gabriel N, El Oud S, et al. The online competition between pro- and anti-vaccination views. Nature 2020. doi:10.1038/s41586-020-2281-1. 32499650

[38] Raws J. A Theory of Justice. Cambridge: Harvard University Press; 1999.

